# Single cell RNA sequencing identifies IGFBP5 and QKI as ciliated epithelial cell genes associated with severe COPD

**DOI:** 10.1186/s12931-021-01675-2

**Published:** 2021-04-06

**Authors:** Xiuying Li, Guillaume Noell, Tracy Tabib, Alyssa D. Gregory, Humberto E. Trejo Bittar, Ravi Vats, Tomasz W. Kaminski, John Sembrat, Mark E. Snyder, Divay Chandra, Kong Chen, Chunbin Zou, Yingze Zhang, Prithu Sundd, John F. McDyer, Frank Sciurba, Mauricio Rojas, Robert Lafyatis, Steve D. Shapiro, Rosa Faner, Toru Nyunoya

**Affiliations:** 1 Department of Medicine, University of Pittsburgh, NW628 UPMC Montefiore, 3459 Fifth Avenue, Pittsburgh, PA 15213 USA; 2 VA Pittsburgh Healthcare System, Pittsburgh, PA USA; 3 Centro Investigación Biomedica en Red (CIBERES), Institut D’investigacions Biomèdiques August Pi I Sunyer (IDIBAPS), Barcelona, Spain; 4 Department of Pathology, University of Pittsburgh, Pittsburgh, PA USA; 5 Vascular Medicine Institute, Department of Medicine, University of Pittsburgh, Pittsburgh, PA USA

**Keywords:** Single cell RNA-seq, COPD, Cigarette smoke

## Abstract

**Background:**

Whole lung tissue transcriptomic profiling studies in chronic obstructive pulmonary disease (COPD) have led to the identification of several genes associated with the severity of airflow limitation and/or the presence of emphysema, however, the cell types driving these gene expression signatures remain unidentified.

**Methods:**

To determine cell specific transcriptomic changes in severe COPD, we conducted single-cell RNA sequencing (scRNA seq) on n = 29,961 cells from the peripheral lung parenchymal tissue of nonsmoking subjects without underlying lung disease (n = 3) and patients with severe COPD (n = 3). The cell type composition and cell specific gene expression signature was assessed. Gene set enrichment analysis (GSEA) was used to identify the specific cell types contributing to the previously reported transcriptomic signatures.

**Results:**

T-distributed stochastic neighbor embedding and clustering of scRNA seq data revealed a total of 17 distinct populations. Among them, the populations with more differentially expressed genes in cases vs. controls (log fold change >|0.4| and FDR = 0.05) were: monocytes (n = 1499); macrophages (n = 868) and ciliated epithelial cells (n = 590), respectively. Using GSEA, we found that only ciliated and cytotoxic T cells manifested a trend towards enrichment of the previously reported 127 regional emphysema gene signatures (normalized enrichment score [NES] = 1.28 and = 1.33, FDR = 0.085 and = 0.092 respectively). Among the significantly altered genes present in ciliated epithelial cells of the COPD lungs, QKI and IGFBP5 protein levels were also found to be altered in the COPD lungs.

**Conclusions:**

scRNA seq is useful for identifying transcriptional changes and possibly individual protein levels that may contribute to the development of emphysema in a cell-type specific manner.

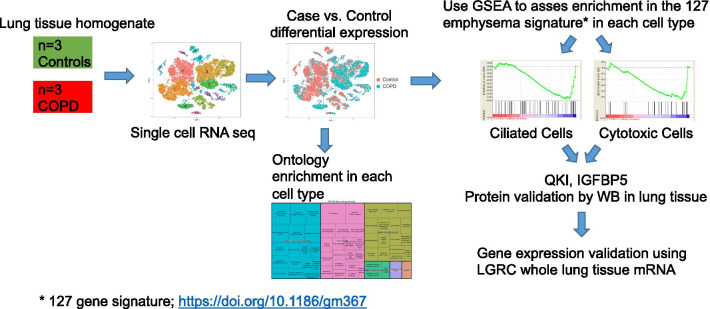

**Supplementary Information:**

The online version contains supplementary material available at 10.1186/s12931-021-01675-2.

## Background

Chronic obstructive pulmonary disease (COPD) is a common respiratory disorder characterized by irreversible expiratory airflow limitation in response to inhalation of noxious stimuli (e.g., cigarette smoke) [1]. COPD is the third leading cause of death [2] and a major economic burden in the United States [3].

COPD is a heterogeneous disorder that can manifest with multiple clinical phenotypes, including emphysema (destructive enlargement of the airspaces distal to terminal bronchioles), chronic bronchitis, and small airway disease [4–7]. Over the last few decades, several lung homogenates and airway transcriptomic studies have been conducted in order to reveal molecular pathways linked to the pathogenesis of smoking-related airflow limitations and emphysema [8–12]. These studies have resulted in the identification of several biological processes, now believed to be associated with smoking-related FEV1 decline [13–15], including (1) chronic immune response and inflammation [16, 17]; (2) imbalance of proteases/anti-proteases [18]; (3) oxidative stress [19, 20]; (4) cellular senescence (permanent loss of proliferative capacity) [21, 22]; and (5) lung epithelial cell (LEC) apoptosis.

In one of the most comprehensive transcriptomic analyses of smoking-related emphysema to date, Campbell, et al*.* profiled gene expression in eight separate regions (based on degree of emphysema) from six emphysematous lungs and compare those transcriptomes with two non-diseased lungs (8 regions × 8 lungs = 64 samples). They identified a total of 127 genes with expression levels significantly correlating with the emphysema severity [23]. Many genes upregulated with increased emphysema severity were involved in inflammation (e.g., the B-cell receptor signaling), while those downregulated with increasing disease severity were implicated in tissue repair (e.g., the transforming growth factor beta (TGFβ) pathway) [23]. This 127 gene emphysema signature was enriched in the transversal studies of lung tissue of patients with severe COPD and emphysema [13, 15]. However, it remains to be elucidated which specific cell types contribute most to this smoking-related emphysematous and small airflow damage transcriptome signature.

Here, we used scRNA-seq technology to identify lung cell-type specific gene expression signatures associated with airflow limitation and/or emphysema. We examined the single-cell transcriptomes of cell populations from lung tissue samples obtained from a representative selection of three ex-smokers with severe COPD/emphysema and three nonsmokers without any history of lung disease. We compared our findings with previously reported whole lung tissue homogenate airflow limitation and emphysema signatures, and experimentally validated the key associated genes.

## Methods

### Human lung tissue samples

For scRNA seq, fresh lung parenchymal tissue samples were obtained from the upper lobes of three nonsmoking subjects without underlying lung disease who underwent warm autopsies and three patients with severe COPD who received lung transplantation (Table 1). For immunoblot analysis (Fig. 2a), frozen lung parenchymal tissue was obtained from smokers without any history of lung disease (n = 5), or very severe COPD (n = 7); both were provided by the University of Pittsburgh, Lung Tissue Research Consortium (LTRC), respectively (Table 2). Data from the Lung Genomics Research Consortium (LGRC) Cohort was used for gene expression analysis of QKI and IGFBP5 (Table 3). The COPD Specialized Center for Clinically Oriented Research (SCCOR) cohort was utilized for comparison of serum IGFBP5 levels between 40 patients with COPD and 40 smokers without clinically evident COPD (Table 4).

**Table 1:** Three normal nonsmokers and three patients with severe COPD

ID	Age	Sex	Smoking (pack-years)	FEV1/FVC	FEV1% ref	DLCO % ref
NL 1	57	F	None	NA	NA	NA
NL 2	18	M	None	NA	NA	NA
NL 3	23	F	None	NA	NA	NA
COPD 1	65	F	60	0.33	33	14
COPD 2	62	F	75	0.29	22	53
COPD 3	61	F	45	0.25	21	20

**Table 2:** Clinical and demographics of the control and COPD subjects (immunoblot analysis)

	Control	COPD (GOLD Stage 4)
Number of subjects	5	7
Age, year, mean (SD)	62.0 (10.0)	62.0 (2.8)
Gender	3 M/2F	4 M/3F
Smoking (pack-years)	35 (10.6)	50.7 (23.1)

**Table 3:** Clinical and demographics of the Lung Genomics Research Consortium (LGRC) Cohort (used for QKI and IGFBP5 gene expression analysis)

	Control	COPD
Number of subjects	108	219
Age, year, mean (SD)	63.6 (11.4)	64.7 (9.7)
Gender (% Female)	55%	43%
Smoking status, (%)		
Never	30%	5.50%
Current	1.90%	6.40%
Ever	58%	86.70%
Unknown	10.10%	1.40%
Pulmonary function, mean (SD)		
FEV1, % predicted	95 (12.6)	50.6 (24.0)
FVC, % predicted	94.4 (13.1)	73,8 (19.5)
DLCO, % predicted	84.1 (16.7)	56.6 (23.1)
Emphysema, % mean (SD)		15.4 (17.1)

*DLCO* diffusing capacity of the lung for carbon monoxide

**Table 4:** Clinical and demographics of the SCCOR Cohort (used for IGFBP5 ELISA)

	Control	COPD
Number of subjects	40	40
Age, year, mean (SD)	66.7 (7.5)	67.5 (6.4)
Gender (% Female)	42.50%	42.50%
Current smoking (%)	47.50%	40%
Pack years (SD)	50.3 (30.5)	65.4 (29.1)
Pulmonary function, mean (SD)		
FEV1, % predicted	99.1 ± 14.7%	70.7 ± 18.8%
FEV1/FVC ratio	0.763 ± 0.037	0.535 ± 0.104
Emphysema %F950^a^	0.005 ± 0.003	0.107 ± 0.086

^a^ % low-attenuation area defined as the fraction of voxels less than − 950 Houndsfield Unit of total voxels identified in regions of the lung

### Preparation of single-cell libraries, sequencing, and analysis

The whole lung tissue samples were processed as described previously [24]. Briefly, lung tissue samples were digested, cell suspensions laded into the Chromium instrument (10× Genomics, Pleasanton, CA), and the resulting barcoded cDNAs were used to construct libraries. RNA-seq was conducted on all mixed samples as a pool. The total number of reads for the three single cell COPD samples was: 456,870,504 reads. Cell-gene specific molecular identifier counting matrices were generated and analyzed using Seurat [25] to identify distinct cell populations [26] and hierarchically clustered using Cluster 3.0 [27].

### Reagents and antibodies

Chemicals were obtained from Sigma Chemical and Calbiochem. Polyvinylidene difluoride membranes were obtained from Bio-Rad. ECL Plus was obtained from Amersham. Antibodies were obtained from various sources: HPRT1 and EPAS1 antibodies were obtained from Cell Signaling; QKI, RTN4, STOM, IGFBP5 and secondary antibodies (horseradish peroxidase-conjugated anti-rabbit or anti-mouse Ig) were obtained from Santa Cruz Biotechnology.

### Immunoblot analysis

Human lung tissues (control and severe COPD groups) were thawed and homogenized in RIPA buffer using a Bullet Blender (next advance). Samples were centrifuged at 4 °C for 10 min at 12,000*G* and resuspended in protein loading buffer. For western blot, about 30 ug of proteins were separated by SDS-PAGE gel and transferred into a nitrocellulose membrane. Membranes were blocked and incubated with primary antibodies and the appropriated secondary antibody HRP conjugated. The signal was acquired with Chemi‐Doc MP (Bio‐Rad) using WesternBright Sirius HRP substrate (advansta).

### ELISA for IGFBP5

The IGFBP5 Human ELISA Kit was purchased from Thermal Fisher Scientific. Serum IGFBP5 levels were measured from 40 control smokers and 40 smokers with COPD according to the vendor’s instructions.

### Gene Ontology enrichment and GSEA

Gene set enrichment analysis (GSEA) was used to identify similarities with previously published emphysema and severity signatures [28]. The gene ontology biological process enrichment was done in R with the ClusterProfiler package [29], and the ontologies were summarized and visualized with the Revigo package [30].

## Results

**Fig. 1:**
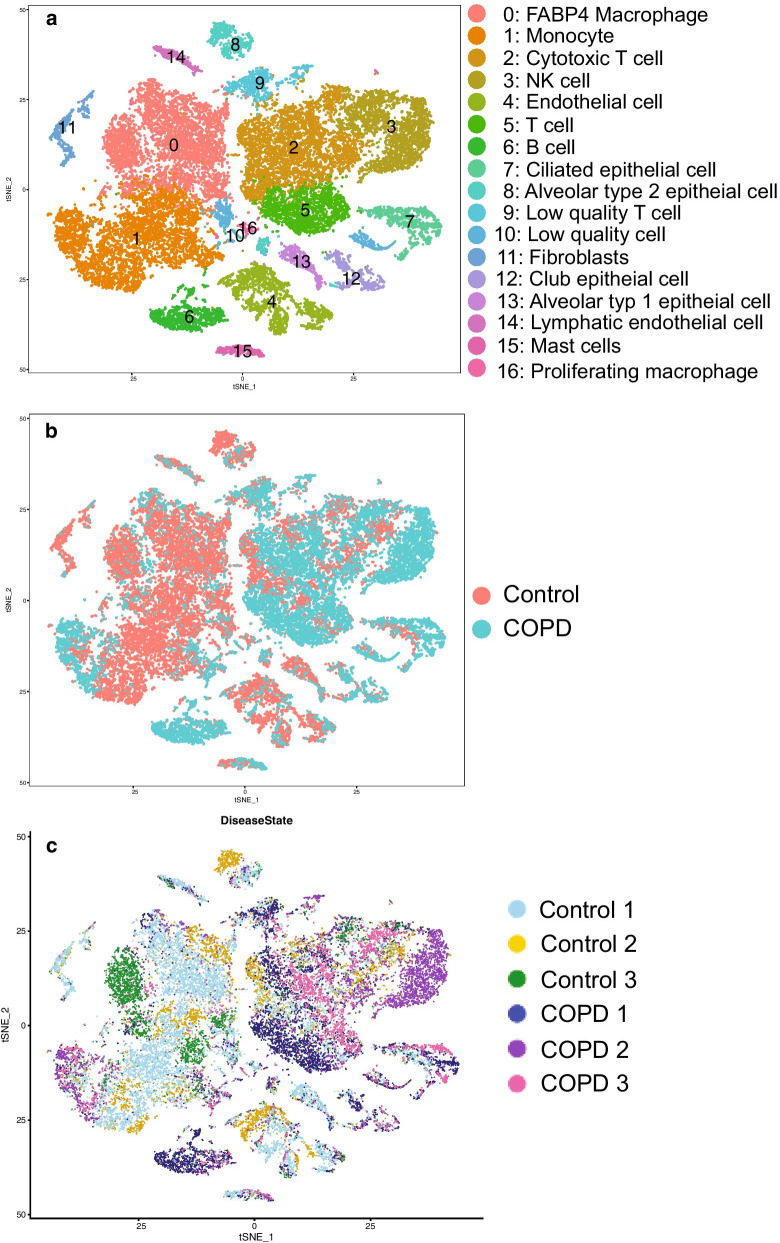

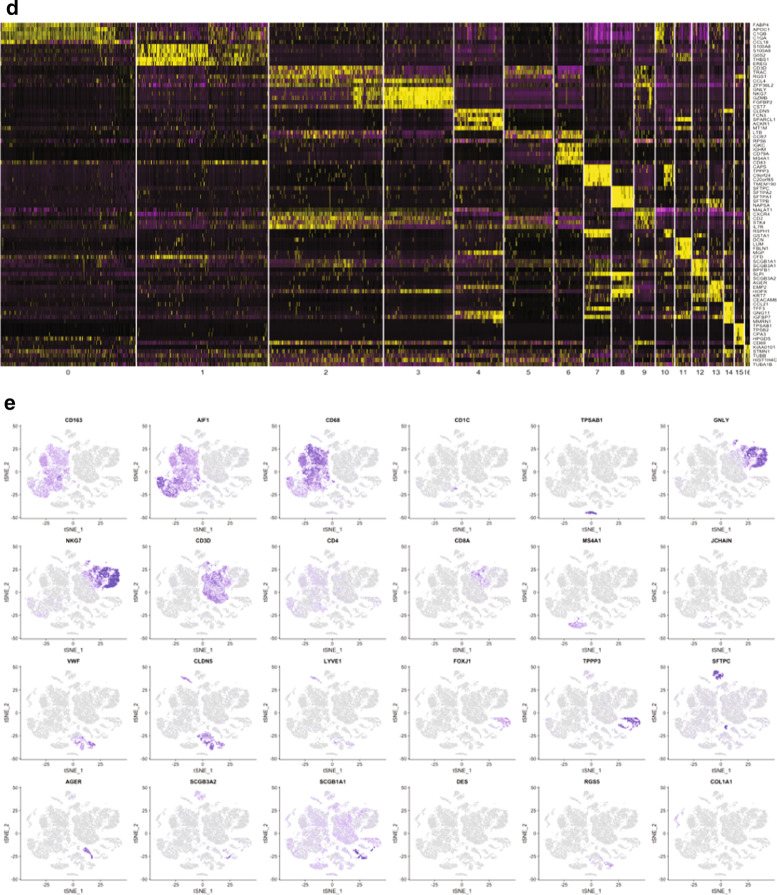
Single cell RNA sequencing reveals 17 distinct cell clusters from human lungs with severe COPD. **a**. scRNA sequencing was conducted using lung parenchymal tissues obtained from three nonsmoking normal subjects and three patients with severe COPD. t-distributed stochastic neighbor embedding (t-SNE) blots were shown using statistically significant principal components and cells were clustered using an unbiased graph-based clustering algorithm (smart local moving [SLM] clustering, which in total identified 17 distinct types of cells, distinguished by color. **b** Cells from disease conditions were indicated by different colors. **c** Cells from individual subjects were indicated by different colors. **d** SLM clustering was made according to distinct gene expression patterns based on various cell types in the lung. **e** SLM clustering is shown graphically in feature plots

**Fig. 2:**
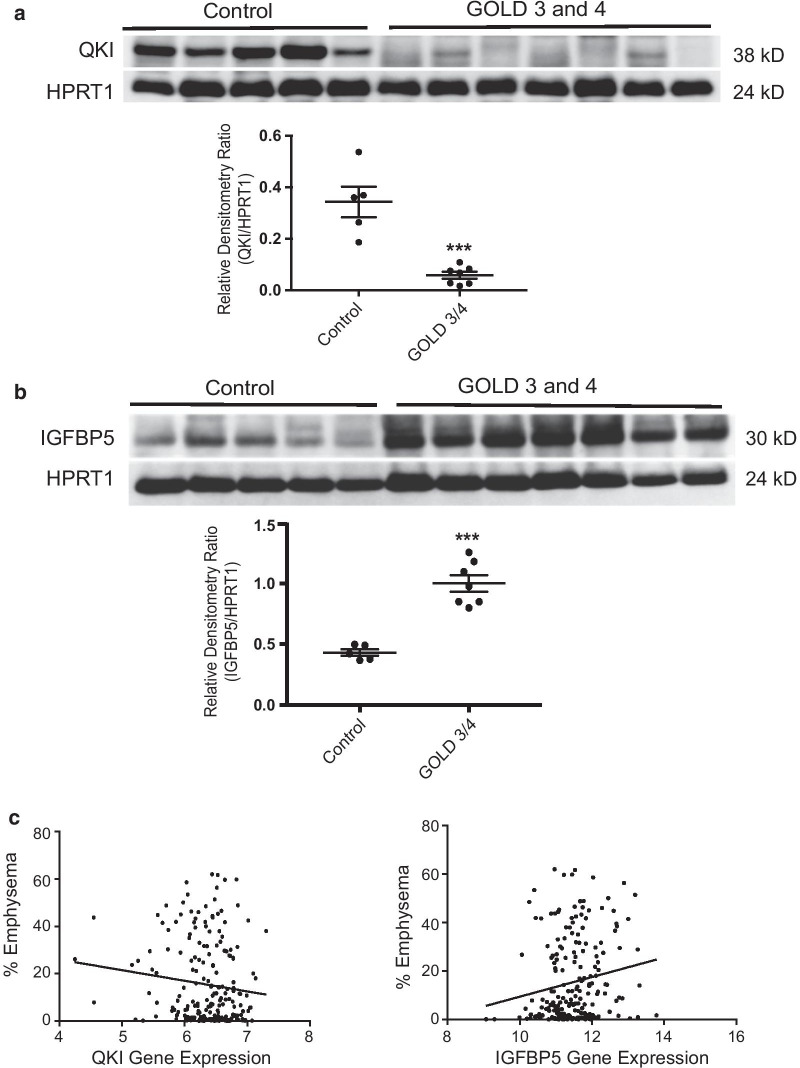
Quantifying protein levels of some genes significantly altered between normal and COPD lungs. **a** Whole parenchymal lung tissues from non-smokers without COPD (n = 5) and former-smokers with COPD GOLD stage 3 or 4 (n = 7) were evaluated for protein expression of QKI by immunoblot analysis. The densitometry data (QKI/HPRT1) obtained from individual groups are expressed as mean ± SEM. ***p value < 0.0001. **b** Whole parenchymal lung tissues from the same groups as in (**a**) were evaluated for protein expression of IGFBP5 by immunoblot analysis. The densitometry data (IGFBP5/HPRT1) obtained from individual groups are expressed as mean ± SEM. ***p value < 0.0001. **c** QKI and IGFBP5 gene expression in whole lung parenchymal tissue obtained from subjects with various severities of emphysema

1. Single cell RNA sequencing reveals 17 distinct cell clusters from human lungs with severe COPD.
  We conducted single cell transcriptomic analysis of all cells obtained from the whole parenchymal lung tissue of three nonsmokers without underlying lung disease and three patients with severe COPD (demographic data: Table 1). Pathology with H&E staining confirmed the presence of moderate to severe emphysematous changes in all three COPD patients (see Additional file 8: Figure S1). We examined a total of 29,961 cells from six subjects (3914–5920 cells/sample). All the samples were pooled and analyzed together to gain the power to detect rare cell types as previously described [24]. t-distributed stochastic neighbor embedding (t-SNE) plots were generated using statistically significant principal components and cells were clustered using an unbiased graph-based clustering algorithm (smart local moving [SLM] clustering), which identified 17 distinct types of cells (Fig. 1a). Cells from disease conditions and the individual subjects were indicated in different colors (Fig. 1b, c, respectively). SLM clustering was performed according to the distinct gene expression patterns based on various cell types in the lung (Fig. 1d). The cell clusters that cannot be reliably classified due to fewer unique molecular identifiers were referred as “low quality” cells. Cell clusters were predominantly identified as: FABP4 as a cell cluster of FABP4 macrophages (#0); S100A8 as a cluster of monocytes (#1); CD3D as cytotoxic T cells (#2); GNLY as NK cells (#3); CLDN5 as endothelial cells (#4); LTB as T cells (#5); IGKC as B cells (#6); CAPS as ciliated epithelial cells (#7); SFTPC as alveolar type 2 epithelial (AT2) cells (#8); MALAT1 as low quality T cells (#9); RSPH1 as low quality cells (#10); DCN as fibroblasts (#11); SCGB1A1 as club epithelial cells (#12); AGER as alveolar type 1 epithelial (AT1) cells (#13); CCL21 as lymphatic endothelial cells (#14); TPSAB1 as mast cells (#15); KIAA0101 as proliferating macrophages (#16). These and other markers provided strong transcriptome signatures for each cell cluster (see Additional file 1: Table S1, shown graphically in feature plots (Fig. 1e). The prevalence of individual cell types between normal nonsmokers and patients with COPD is shown in Table 5. None of the differences in the abundance of individual cell types between normal nonsmokers and COPD patients reached statistical significance (Wilcoxon). However, a trend existed for decreased percentages of macrophages, endothelial cells, AT2 cells, and fibroblasts in the COPD lungs.
2. Gene expression in 17 individual types of cells in severe emphysematous lungs.
  For each cluster, the differential gene expression among the cells of the three COPD patients vs. the controls was computed using a Wilcoxon test. This analysis identified the number of differentially expressed genes per cluster when applying the filter of log fold change >|0.4| and FDR = 0.05 (Additional file 2: Table S2). Interestingly, the major populations showing the largest differences in gene expression were: monocytes, macrophages, low quality cells, ciliated epithelial cells, T cells, AT2 cells, and NK cells (Table 5). Most gene expression changes corresponded to an increased transcription (up-regulation) in patients with COPD (Table 5).
3. The Gene Ontology enrichment Gene expression in 17 individual types of cells from severe emphysematous lungs.
  Next, we performed the functional enrichment with the upregulated or downregulated genes in COPD for each population for GO biological processes (Additional file 3: Table S3 and Additional file 4: Table S4). Results for the upregulated genes in COPD (i.e., macrophages, monocytes, ciliated epithelial cells and NK cells), have been summarized with Revigo and are shown as Treemaps (Additional file 8: Figures S2–S5). Of note, these four individual cell types shared ontologies related to the activation of T cells, defense response and three of them (not macrophages) also presented ontologies related to the viral life cycle.
4. Single cell contribution to the 127 emphysema-related gene signatures.
  Next, we identified how the transcriptomic changes of each cell type contribute to the previously described 127 gene signatures of emphysema reported by Campbell et al. [23]. We computed the overlap using GSEA (Table 6). Three individual cell types were enriched with a nominal p value < 0.05 and an FDR < 0.1 (i.e., ciliated epithelial cells, cytotoxic T cells, and low quality T cells). The genes in the core enrichment for these three individual cell types and the values of differentially expressed genes in Campbell’s data set are shown in Additional file 5: Table S5. EPAS1, QKI and STOM were differentially expressed core genes in all three-cell types (Additional file 1: Table S5).
  As a complementary analysis, we showed which individual cell types differentially expressed the 127 emphysema related genes (Additional file 6: Table S6 and Additional file 8: Figure S6). FCN3, RTN4 and CCR7 were differentially expressed in a total of five individual cell types, and EPAS1, QKI and STOM in the four individual cell types. The individual cell types with more differentially expressed genes were: monocytes (n = 10), AT2 cells (n = 9), macrophages (n = 8), ciliated cells (n = 5), and T cells (n = 5).
5. Comparison with severe airflow limitation genes.
  Next, we determined whether the gene expression changes observed in the individual cell types represent the previously identified gene signatures in whole lung tissue of patients with severe airflow limitation. To achieve this, we assessed the enrichment with GSEA of the individual cell types with the differentially expressed genes between n = 17 non-smokers and n = 30 patients with GOLD stage 4 obtained from LTRC (GSE47460, GPL14550). Overall, the enrichment in all individual cell types appeared to be consistent with the genes differentially expressed in whole lung tissue. However, the enrichment was significant at a nominal p value for only four individual cell types (mast cells, proliferating macrophages, monocytes, and FBPB4 macrophages) (Table 7). The full list of genes differentially expressed in the LTRC and differentially expressed in the individual cell clusters is shown in Additional file 7: Table S7.
  Our analysis identified several genes expressed in a distinct cell types that were previously reported to be differentially expressed in lung tissue homogenates according to the severity of airflow limitation [14, 31]. These genes are FGG (AT2 cells), CCL19 (monocytes), PLA2G7 (macrophages), HP (macrophages), TNFSF13B (monocytes) and FCRLA (B cells). In addition, following genes were differentially expressed in several individual cell types: S100A10 (n = 7), RPS10, GNG11, and CAV1 (n = 6), S100A6 (n = 5), and AGER (n = 4).
6. Quantifying protein levels of some genes significantly altered between normal and COPD lungs.
  To determine whether significantly altered genes between normal and COPD lungs also correlate with the related protein levels, whole parenchymal lung tissues from non-smokers without COPD (*n* = 5 per group) and former-smokers with COPD GOLD stage 3 or 4 (*n* = 6 or 7 per group) were evaluated for protein expression of QKI, STOM, and EPAS1 by immunoblot analysis. We also included IGFBP5 (insulin-like growth factor binding protein 5), which was primarily expressed in three-cell types (ciliated cells, fibroblasts, and lymphatic endothelial cells). Although IGFBP5 was significantly upregulated in only ciliated cells of COPD lungs relative to controls (p = 0.029), given a known genetic association of IGFBP5 with COPD [32] and a potential application as a serum biomarker in patients with COPD, we added IGFBP5 to this analysis.
  Consistent with altered gene expression, QKI and IGFBP5 protein levels were significantly increased in the COPD lungs relative to non-smokers (Fig. 2a, b), but neither STOM nor EPAS1 protein levels were altered (Additional file 8: Figure S7A). Further, we determined whether QKI and IGFBP5 gene expressions in whole lung tissue correlated with emphysema severity. QKI expression appeared to decrease according to % emphysema (n = 208; p = 0.0854), whereas, IGFBP5 expression significantly increased according to % emphysema (p = 0.0150). Since IGFBP5 is an excretory protein, we measured the serum levels of IGFBP5 in smokers with or without COPD (n = 40, each group), but detected no significant differences between the two group (Additional file 8: Figure S7B). These results suggest that some of the significantly altered genes in COPD lungs identified by scRNA seq indeed correlate with the individual protein levels in the whole lung tissue.

**Table 5:** Identified clusters, number of differentially expressed genes in case vs. control, mean % of cells belonging to this cluster both in cases and controls, fold change differences between cluster percentage and the p value of the comparison of the cluster frequency in cases vs. controls both with t-test and Wilcoxon-test

Cluster	Population	# DE genes	Up	Down	Control % of cells	*COPD* % of cells	Fold change	p value (t.test)	p value (Wilcox)
0	FABP4 Macrophages	868	620	248	27.97 ± 10.74	5.15 ± 1.57	5.43	0.02	0.057
1	Monocytes	1499	677	822	23.80 ± 12.90	11.24 ± 4.84	2.12	0.18	0.23
2	Cytotoxic T cells	539	165	374	11.18 ± 6.48	21.86 ± 9.80	− 1.96	0.14	0.11
3	NK cells	431	233	198	3.85 ± 2.89	22.14 ± 27.41	− 5.75	0.23	0.4
4	Endothelial	242	185	57	9.17 ± 3.90	2.37 ± 0.52	3.87	0.03	0.057
5	T cells	213	64	149	2.11 ± 1.00	11.68 ± 9.02	− 5.54	0.08	0.23
6	B cells	153	30	123	0.99 ± 0.59	6.80 ± 7.95	− 6.84	0.19	0.23
7	Ciliated	590	433	157	1.84 ± 0.98	6.18 ± 4.36	− 3.35	0.10	0.23
8	AT2	476	34	442	4.12 ± 2.52	0.96 ± 0.32	− 4.28	0.09	0.057
9	Low quality T cells	41	14	27	1.75 ± 0.68	3.71 ± 2.07	− 2.12	0.13	0.11
10	Low quality cells	596	369	227	2.02 ± 0.95	2.83 ± 1.50	− 1.40	0.41	0.63
11	Fibroblasts	96	87	9	3.17 ± 1.62	0.27 ± 0.07	11.60	0.03	0.057
12	Club cells	98	70	28	1.35 ± 0.81	2.72 ± 2.16	− 2.02	0.29	0.63
13	AT1	68	59	9	2.60 ± 1.91	0.46 ± 0.36	5.71	0.12	0.11
14	Lymphatic-endothelial	27	20	7	1.93 ± 1.10	0.33 ± 0.29	5.85	0.06	0.23
15	Mast cells	32	25	7	1.32 ± 0.79	0.91 ± 0.24	1.44	0.44	0.63
16	Proliferative macrophages	111	55	56	0.84 ± 0.35	0.39 ± 0.25	2.17	0.12	0.11

**Table 6:** GSEA enrichment of the different clusters with the 127 gene signature

Cluster	ES	NES	NOM p-val	FDR q-val	FWER p-val
Ciliated	0.33	1.33	0.00	0.09	0.00
Cytotoxic T	0.33	1.29	0.00	0.09	0.00
Low quality T cells	0.47	1.29	0.00	0.11	0.00
AT2	0.39	1.27	0.09	0.18	0.04
Fibroblasts	0.40	1.31	0.10	0.18	0.05
T cells	0.31	1.17	0.10	0.21	0.05
Proliferative macrophages	0.37	1.23	0.10	0.22	0.05
B cells	− 0.24	− 0.97	0.18	0.28	0.09
NK	0.31	1.05	0.29	0.37	0.14
AT1	0.33	1.00	0.43	0.52	0.22
Monocytes	0.20	0.84	0.68	0.77	0.36
Macrophages	− 0.22	− 0.92	0.70	0.83	0.37
Club	− 0.20	− 0.82	0.76	0.88	0.39
Low quality	− 0.25	− 0.78	0.88	1.00	0.43
Endothelial	− 0.17	− 0.53	0.89	1.00	0.44
Mast cells	0.28	0.77	0.92	1.00	0.47

**Table 7:** GSEA enrichment results of the different clusters with the differential gene expression of the LTRC (COPD Gold 4 vs. non-smokers)

	Size	ES	NES	NOM p-value	FDR q-value	FWER p-value
Mast cells	70	− 0.62	− 1.72	0.00	0.07	0.04
Proliferating macrophages	164	− 0.56	− 1.71	0.01	0.04	0.05
Monocytes	103	− 0.63	− 1.58	0.03	0.10	0.12
FBPB4 macrophages	27	− 0.61	− 1.46	0.04	0.15	0.23
AT2	80	− 0.56	− 1.56	0.06	0.09	0.14
B cells	48	− 0.47	− 1.42	0.07	0.14	0.28
Club	83	− 0.51	− 1.38	0.09	0.16	0.32
Fibroblasts	245	− 0.49	− 1.45	0.12	0.13	0.24
Cytotoxic T cells	30	− 0.54	− 1.33	0.13	0.16	0.39
Low quality T cells	126	− 0.45	− 1.38	0.13	0.14	0.33
Ciliated	55	− 0.48	− 1.17	0.30	0.30	0.57
T cells	36	− 0.42	− 1.14	0.32	0.30	0.60
Endothelial	94	− 0.36	− 0.93	0.53	0.51	0.76
AT1	221	− 0.12	− 0.56	0.97	0.99	0.92
NK cells	23	− 0.21	− 0.55	0.97	0.93	0.92

## Discussion

This study uses scRNA seq from human lung homogenate in order to identify the specific cell types driving gene expression changes found in bulk RNA sequencing of patients with severe emphysema and airway obstruction. We found that: (1) t-SNE and clustering of scRNA-seq data identified a total of 17 distinct populations based on predetermined markers per cell type. Monocytes, macrophages, ciliated cells and low quality cells exhibited more differentially expressed genes in cases vs. controls relative to the other cell types; (2) GSEA revealed that the populations contributing most to the previously reported emphysema signature were ciliated cells, cytotoxic T cells and low quality T cells. While in the severe COPD LTRC signature, the populations enriched by GSEA were proliferating macrophages, mast cells, AT2 cells and monocytes; (3) key COPD associated genes were found to be expressed by specific cell types:. FGG (AT2 cells), CCL19/TNFSF13B (monocytes) and PLA2G7 (macrophages); (4) We verified the expression of some of the specific scRNA seq differentially expressed genes at the protein level as well (i.e. QKI and IGFBP5).

### Previous studies

Although the scRNA seq methodology has been applied to the profiling of lung tissue of patients with IPF [33–35], this is the first study to our knowledge which profiles the lung tissue of patients with COPD. In relation to severe COPD and emphysema, several studies have reported the transcriptomic profile of lung tissue homogenates [14, 31], but the scRNA seq has several advantages over the previously used RNA seq methods. First, scRNA seq can determine which cell types are responsible for the significant transcriptomic changes in a disease process (e.g., the emphysematous and/or airflow limitation signature). Second, scRNA seq can detect alteration of the cell composition as opposed to the whole cell RNA-seq. Third, scRNA seq may uncover an important molecular pathway unique to a specific cell type that contributes to the development of the disease.

### Interpretation of novel findings

We identified 17 cell subtypes in the lung tissue of patients with severe COPD and non-smoking controls. In relation to cell composition, differences were not statistically significant, but in agreement with previous reports. We observed an overall increase of immune cell types (T, NK and B cells) and a decrease in structural cells (Fibroblasts, AT2 cells and endothelial cells) [36]. However, the sampling effects inherent to scRNA seq may have contributed to the skewed proportion of distinct cell populations. Interestingly, we found that the genes up-regulated in the cell types with more differential expression (monocytes, macrophages, ciliated cells, cytotoxic T cells and AT2 cells) were related to T cell activation, antigen presentation and signaling. The role of cytotoxic T cells (CD8^+^) in severe COPD has long been recognized [36–38] and emphysema has been proposed to be associated with a Th1 response activated by infiltrating ILC1, NK, and LTi cells [39]. Here, we expand these findings by showing that antigen presenting cells (macrophages, monocytes and AT2 cells) are also involved in T cell activation. Interestingly, the viral related ontologies appeared to be enriched in these cell types as well as in ciliated epithelial cells and NK cells. Further work profiling the virome in parallel with cellular phenotyping may complement these findings.

In relation to the previously described 127 emphysema gene signature, our GSEA analysis showed an enrichment of genes differentially expressed by ciliated and T cells (cytotoxic and of low quality), suggesting an active involvement of T cells in the emphysematous tissue remodeling and accumulation of primary cilia [39, 40]. Some of the genes associated with the homing of B cells and previously identified by homogenate tissue profiling (i.e. CCL19, TNFSF13B) [23, 31] were found in the current study to be expressed by macrophages and monocytes. Yet in the current analysis, the increase in B cells was observed in 2 of the 3 severe COPD samples, and the genes hyper expressed by the severe COPD B cells were related to the T cell activation in concordance with previous works [31]. Upregulation of gene expression for fibrinogen (FGG) is a well-recognized biomarker in COPD [41] and is produced by AT2 cells. Fibrinogen has a well-known role in both innate and T cell mediated adaptive immune responses to bacteria [42]. Similar to these previous findings, our analysis showed an upregulation of genes related to the antigen presentation in AT2 cells, suggesting a role in the stimulation and perpetuation of the observed immune response in the lung. AGER, another well-known gene associated with COPD [43], was upregulated in four cell types in our analysis (macrophages, monocytes, low quality T cells and B cells) that are associated with immune response, suggesting a role of the RAGE axis in the chronic immune infiltrate observed in severe COPD/emphysema. In our analysis, club cells that express the COPD associated CC-16 protein, were found to have altered mRNA catabolism and toxic substance response pathways. Further investigation is warranted to determine the impact of these alterations in cell functionality [44].

Finally, in this study, we attempted to verify whether differences in gene expression correlated with differential protein content in the cellular populations derived from COPD and normal lungs. Among several common targets, we found that COPD lungs exhibit decreased protein levels of QKI and increased protein levels of IGFBP5.

Our scRNA seq data show that QKI is expressed abundantly in myeloid cells, endothelial cells, and AT1 cells, whereas IGFBP5 is expressed in ciliated cells, fibroblasts, and lymphatic endothelial cells relative to the other types of cells. QKI, a KH domain containing RNA binding protein, regulates versatile mRNA metabolism—splicing, export, stability, and protein translation [45]. Loss-of-function mutations in QKI disturb myelination and cause embryonic lethality [46]. QKI has been implicated in various disease processes, including atherosclerosis [47], tumorigenesis [48], and fibrosis [49]. IGFBP5, insulin-like growth factor binding protein 5, is one of the six proteins of the IGFBP family [50]. IGFBP proteins bind IGF-I/II and regulate their bioavailability and downstream signaling. In addition, IGFBP proteins can regulate cell growth and survival independent of IGF-I/II [50]. In particular, IGFBP5 plays a causal role in the induction of cellular senescence and inflammation [51], which may be linked to pulmonary fibrosis [52]. Further, an intergenic SNP of IGFBP5 (rs6435952) associates with airway obstruction [32]. Although IGFBP5 is a secretory protein [53], there was no significant change in the serum levels of IGFBP5 in COPD patients compared with control smokers. However, there may be excretory impairment of IGFBP5 protein in the COPD lung which remains to be determined in future studies. An in vivo animal study will be necessary to elucidate causal roles for QKI and IGFBP5 in the development of smoking-induced COPD.

## Limitations

Several scRNA seq studies using the human lung tissue were conducted and profiled approximately 3000 to 6000 per sample [35, 54, 55]. The range of cell number per sample analyzed in this study are pretty comparable to the previous studies. However, the main limitation of this study is the sample size as we have analyzed three control subjects without underlying lung disease and three patients with severe COPD. We used the nonsmoking control libraries which were included in another dataset we published [35]. Two and one of the control subjects did not match with respect to age and sex with the COPD subjects, respectively. The potential effects of age and sex differences between the control and COPD subjects on the data analysis could be substantial; however, it is difficult to determine as a result of the small sample size used in this study.

Active cigarette smoke exposure has the major influence on gene expression [56]. Furthermore, it is known that smoke dose exposure and duration and length of cessation also affect gene expression. Accordingly, to control for an active smoking effect, included in the three COPD subjects are a former smoker who had stopped smoking for at least 6 months. Due to this, there was no significant increase either in the ALDH3A1 or CYP1A1 genes (known transcriptional targets of the aryl hydrocarbon receptor robustly upregulated by CS [56]) in the lungs of COPD subjects relative to the controlled nonsmoking subjects. In addition, our findings are not representative of the COPD heterogeneity and we will need to increase the sample size with appropriately controlled smoking subjects (i.e., matching age, sex, smoking dose and time and cessation period) to address this open issue in future investigations. Notwithstanding this limitation, the main focus of this work has been to use the generated data to explore which cell types express the key genes previously shown to be associated with COPD and emphysema.

## Conclusions

We identified ciliated and CD8^+^ T cells as prominent cell types associated with the 127 gene signature associated with emphysema. Our findings support a prominent role of the immune response in severe COPD, with the implication of structural and antigen presenting cells in its homing and perpetuation. Finally, QKI and IGFBP5 are identified as potential COPD biomarkers, whose both gene and protein expression are significantly altered in COPD lungs relative to normal lungs. The causal role of QKI and IGFBP5 in the development of COPD/emphysema will need further investigation.

## Supplementary Information

**Additional file 1: Table S1.** Transcriptomic markers per cell type (top 100 genes per cluster).

**Additional file 2: Table S2.** DE genes per cell type, log FC|0.4| and FDR.

**Additional file 3: Table S3.** Biological process gene ontology enrichment in genes upregulated in each cluster in COPD.

**Additional file 4: Table S4.** Biological process gene ontology enrichment in genes downregulated in each cluster in COPD.

**Additional file 5: Table S5.** Genes present in the GSEA core enrichment with the 127 emphysema gene signature.

**Additional file 6: Table S6.** Genes from the 127 gene signature differentially expressed by cell type.

**Additional file 7: Table S7.** Genes from the sever COPD signature (LTRC) differentially expressed by cell type.

**Additional file 8: Figure S1.** Lung histology of three COPD cases (hematoxylin & eosin staining). **Figure S2.** Revigo summary of biological processes enriched in Macrophages. **Figure S3.** Revigo summary of biological processes enriched in Monocytes. **Figure S4.** Revigo summary of biological processes enriched in Ciliated epithelial cells. **Figure S5.** Revigo summary of biological processes enriched in NK cells. **Figure S6.** Differentially expressed genes in the 127 gene signature per type of cell, per patient. **Figure S7.** A: Immunoblot analysis for STOM, EPAS1, RTN4 (controls vs. COPD GOLD stage 4). B: Serum IGFBP5 measurements in controls (n = 40) and COPD cases (n = 40).

## Data Availability

All data generated or analyzed during this study are included in this published article [and its additional information files].

## Abbreviations

AGER: Advanced glycosylation end product receptor
AT1: Alveolar type 1 epithelial
AT2: Alveolar type 2 epithelial
CAPS: Calcyphosine
CC-16: Club cell protein 16
CCL19: C–C motif chemokine ligand 19
CCL21: C–C motif chemokine ligand 21
CCR7: C–C motif chemokine receptor 7
CLDN5: Claudin 5
COPD: Chronic obstructive pulmonary disease
CS: Cigarette smoke
DCN: Decorin
EPAS1: Endothelial PAS domain protein 1
ECL: Enhanced luminol-based chemiluminescent
ELISA: Enzyme-Linked ImmunoSorbent Assay
FABP4: Fatty acid binding protein 4
FCN3: Ficolin 3
FCRLA: Fc receptor like A
FDR: False discovery rate
FEV1: Forced expiratory volume in 1 s
FGG: Fibrinogen gamma chain
GNG11: G protein subunit gamma 11
GNLY: Granulysin
GO: Gene Ontology
GOLD: Global Initiative for Chronic Obstructive Lung Disease
GSEA: Gene set enrichment analysis
LTB: Lymphotoxin beta
H&E: Hematoxylin and eosin
HP: Haptoglobin
HPRT1: Hypoxanthine phosphoribosyltransferase 1
HRP: Horseradish peroxidase
IGFBP5: Insulin-like growth factor binding protein 5
IGKC: Immunoglobulin Kappa Constant
ILC1: Innate lymphoid cell 1
IPF: Idiopathic pulmonary fibrosis
LEC: Lung epithelial cell
LGRC: Lung Genomics Research Consortium
LTi: Lymphoid tissue inducer
LTRC: Lung Tissue Research Consortium
MALAT1: Metastasis Associated Lung Adenocarcinoma Transcript 1
NK cell: Natural killer cell
PLA2G7: Phospholipase A2 Group VII
QKI: KH domain containing RNA binding protein
RAGE: Receptor for advanced glycation endproducts
RIPA: Radioimmunoprecipitation assay
RPS10: Ribosomal protein S10
RSPH1: Radial spoke head component 1
RTN4: Reticulon 4
S100A6: S100 calcium binding protein A6
S100A8: S100 calcium binding protein A8
S100A10: S100 calcium binding protein A10
SCCOR: COPD specialized center for clinically oriented research
SCGB1A1: Secretoglobin Family 1A Member 1
scRNA seq: Single-cell RNA sequencing
SDS-PAGE: Sodium dodecyl sulfate-polyacrylamide gel electrophoresis
SFTPC: Surfactant protein C
SLM: Smart local moving
SNP: Single nucleotide polymorphism
STOM: Stomatin
TGFβ: Transforming growth factor beta
TNFSF13B: TNF superfamily member 13b
TPSAB1: Tryptase alpha/beta 1
t-SNE: T-distributed stochastic neighbor embedding
