# Supplementary material for: Single cell RNA sequencing identifies IGFBP5 and QKI as ciliated epithelial cell genes associated with severe COPD
Source: Respir Res. 2021 Apr 6;22:100. doi: 10.1186/s12931-021-01675-2 (PMC8022543; doi:10.1186/s12931-021-01675-2)
Supplement: Supplementary file 8 — Additional file 8: Figure S1. Lung histology of three COPD cases (hematoxylin & eosin staining). Figure S2. Revigo summary of biological processes enriched in Macrophages. Figure S3. Revigo summary of biological processes enriched in Monocytes. Figure S4. Revigo summary of biological processes enriched in Ciliated epithelial cells. Figure S5. Revigo summary of biological processes enriched in NK cells. Figure S6. Differentially expressed genes in the 127 gene signature per type of cell, per patient. Figure S7. A: Immunoblot analysis for STOM, EPAS1, RTN4 (controls vs. COPD GOLD stage 4). B: Serum IGFBP5 measurements in controls (n = 40) and COPD cases (n = 40). [file 12931_2021_1675_MOESM8_ESM.pdf]

Lung Histology of three COPD cases (Hematoxylin & Eosin Staining)

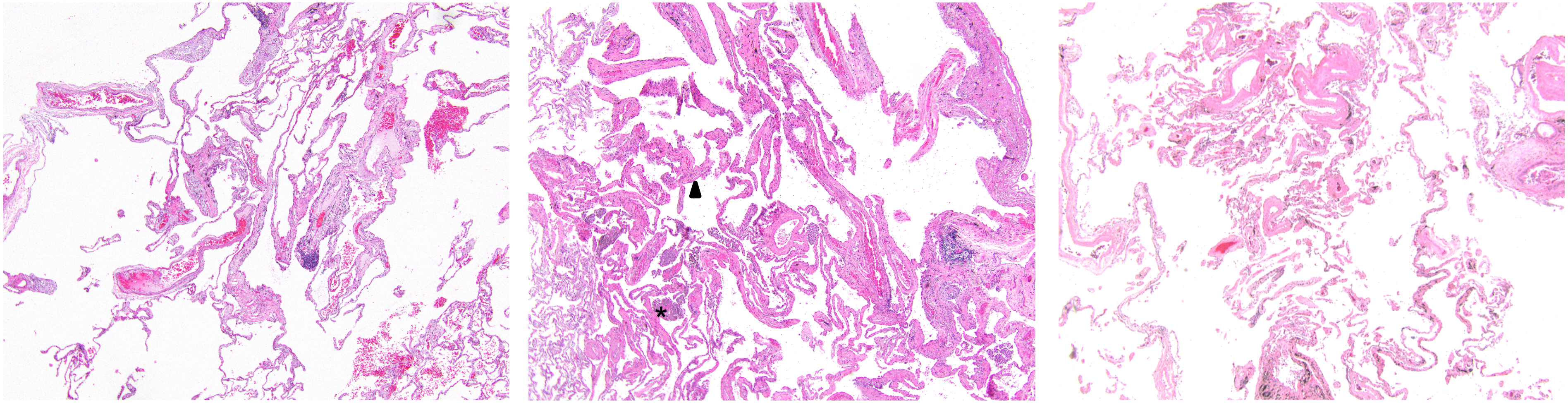

Case 1 Case 2 Case 3

Cases 1 and 3: Left upper lobe histologic sections show extensive centrilobular emphysema manifesting as loss of the normal alveolar architecture with simplification of the airspaces (Cases 1 and 3). Case 2: Left upper lobe histologic section shows similar findings to Cases 1 and 3. In addition, there is extensive dust-laden (smoker's) macrophages (asterisk) in the airspaces and thickening of the alveolar septum by collagenous fibrosis (arrowhead) indicating smoking-related interstitial fibrosis (Magnification 40x).

Figure S1

Macrophages: Revigo summary of GO.BP enriched in upregulated COPD genes

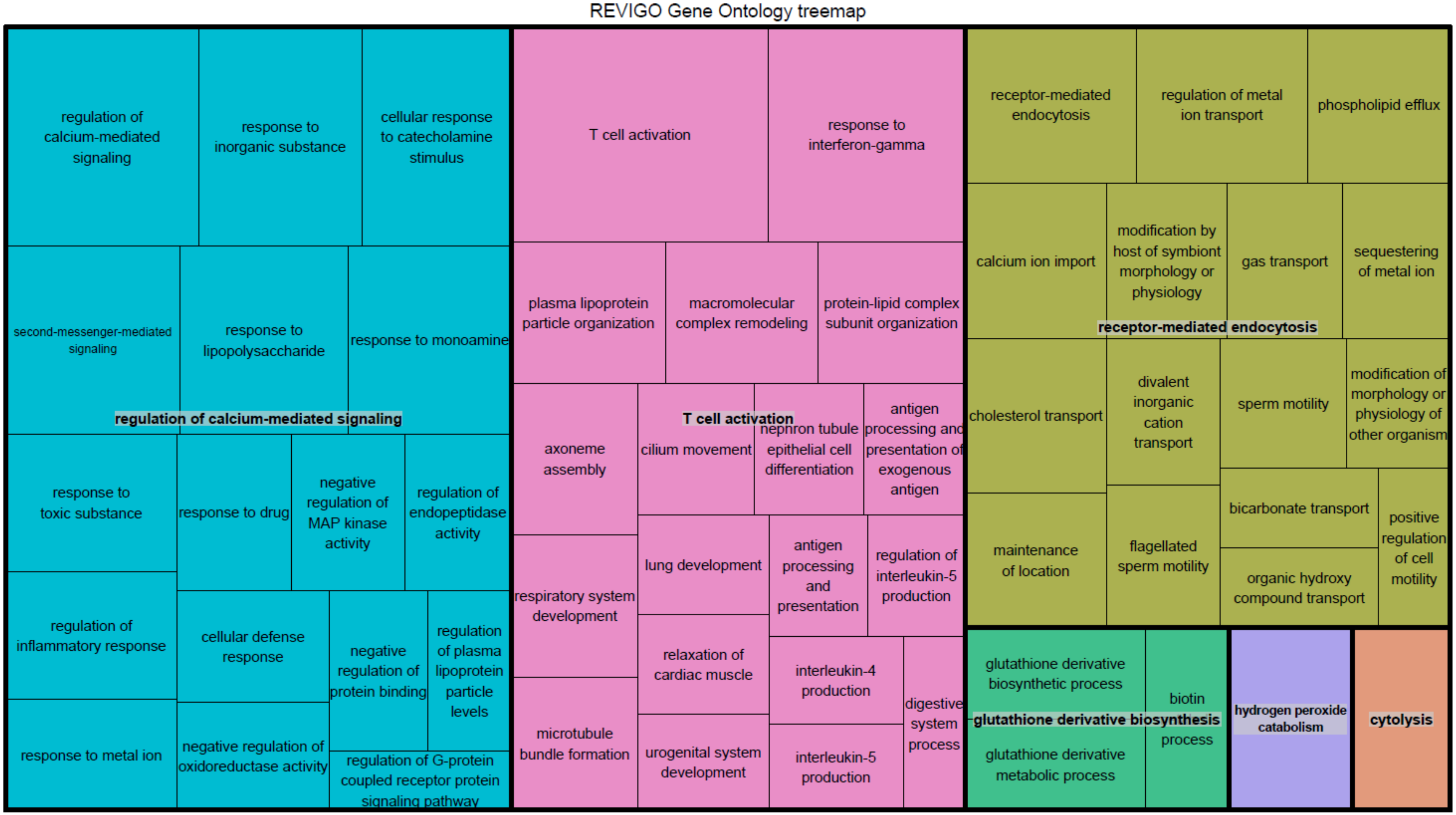

Figure S2

Monocytes: Revigo summary of GO.BP enriched in upregulated COPD genes

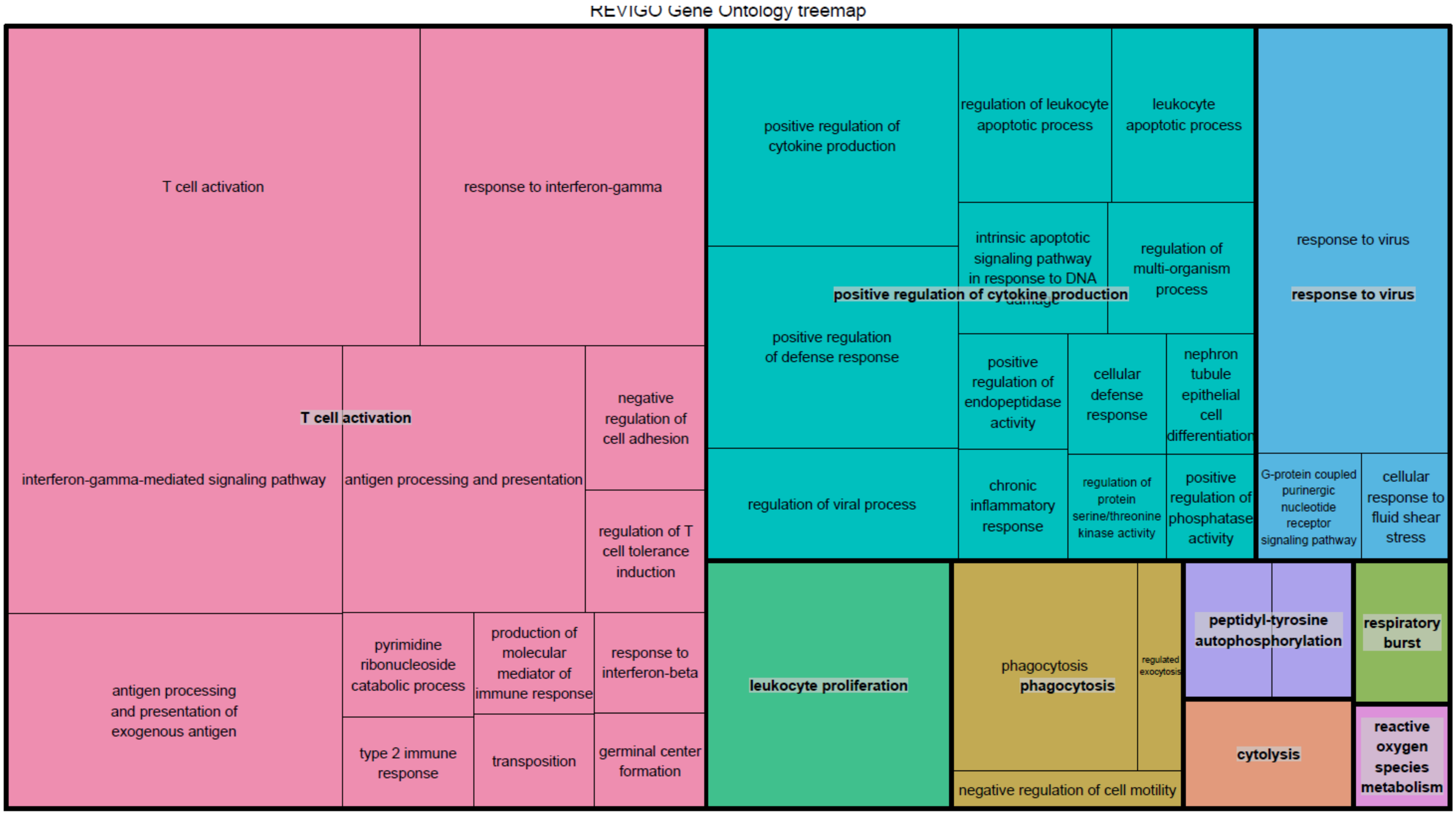

Figure S3

Ciliated epitelial cells: Revigo summary of GO.BP enriched in upregulated COPD genes

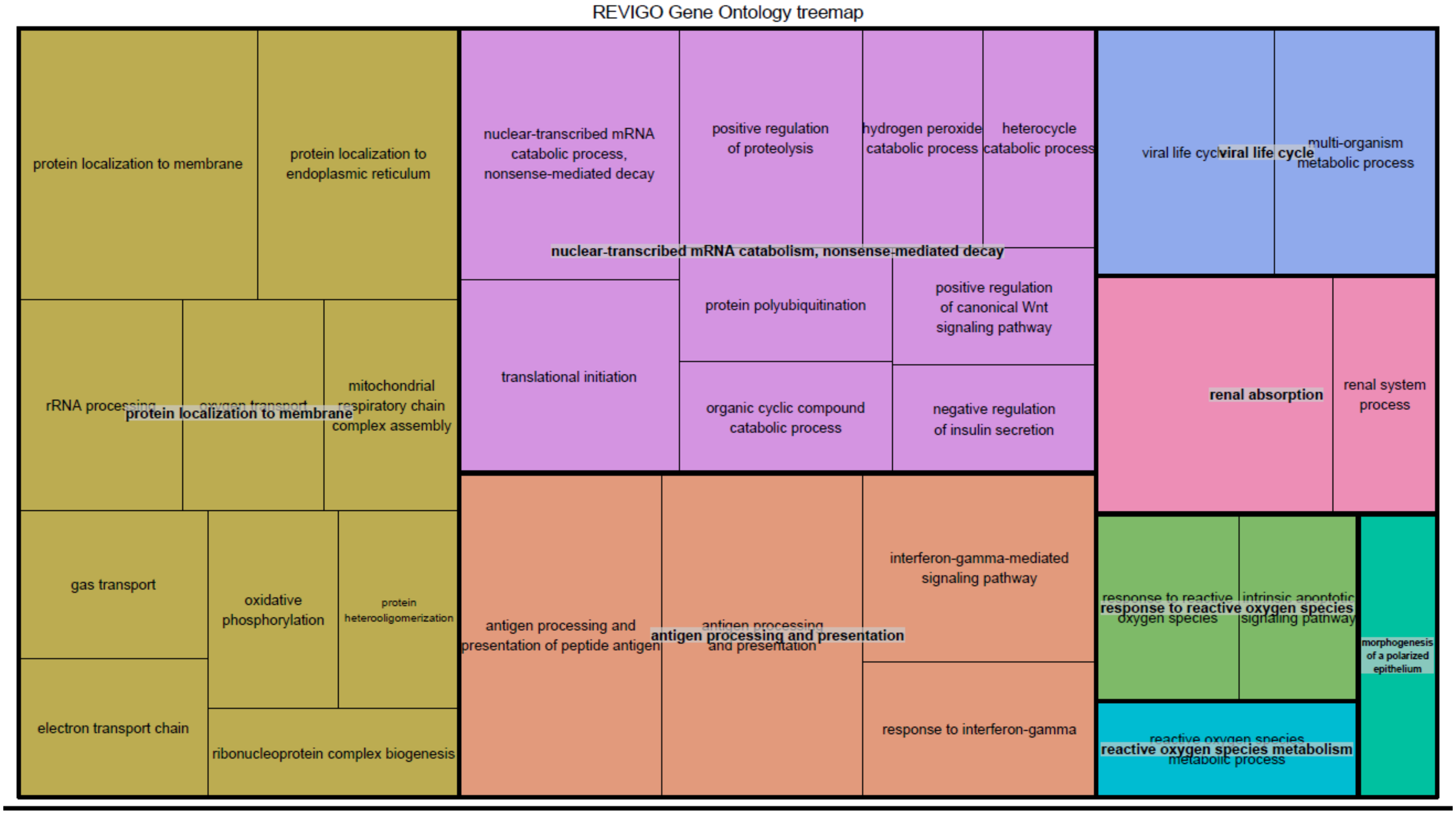

Figure S4

NK cells: Revigo summary of GO.BP enriched in upregulated COPD genes

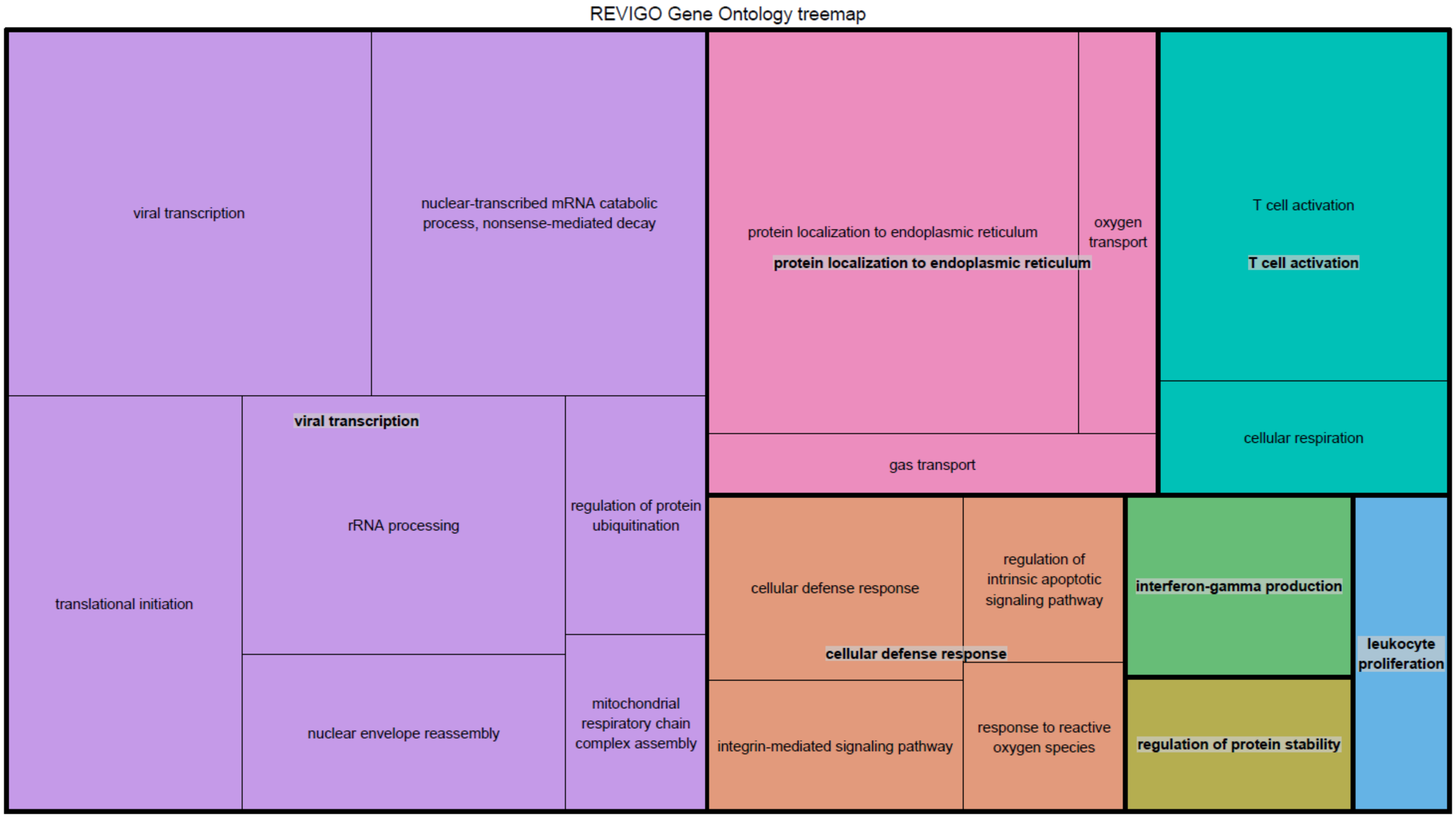

Figure S5

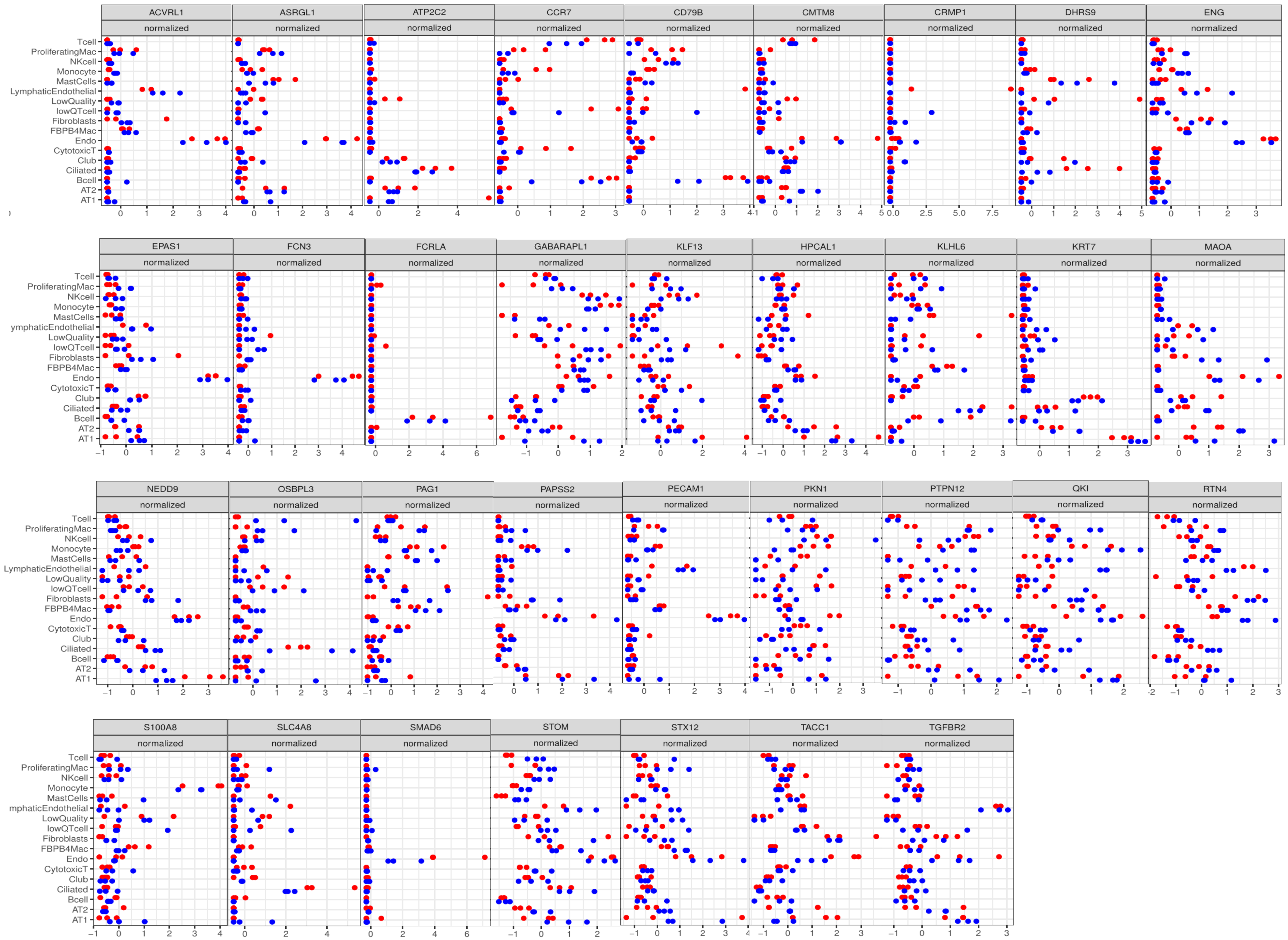
ACVRL1
ASRGL1
ATP2C2
CCR7
CD79B
CMTM8
CRMP1
DHRS9
ENG
EPAS1
FCN3
FCRLA
GABARAPL1
KLF13
HPCAL1
KLHL6
KRT7
MAOA
NEDD9
OSBPL3
PAG1
PAPSS2
PECAM1
PKN1
PTPN12
QKI
RTN4
S100A8
SLC4A8
SMAD6
STOM
STX12
TACC1
TGFBR2
normalized
Tcell
ProliferatingMac
NKcell
Monocyte
MastCells
LymphaticEndothelial
LowQuality
lowQTcell
Fibroblasts
FBPB4Mac
Endo
CytotoxicT
Club
Ciliated
Bcell
AT2
AT1

Figure S6

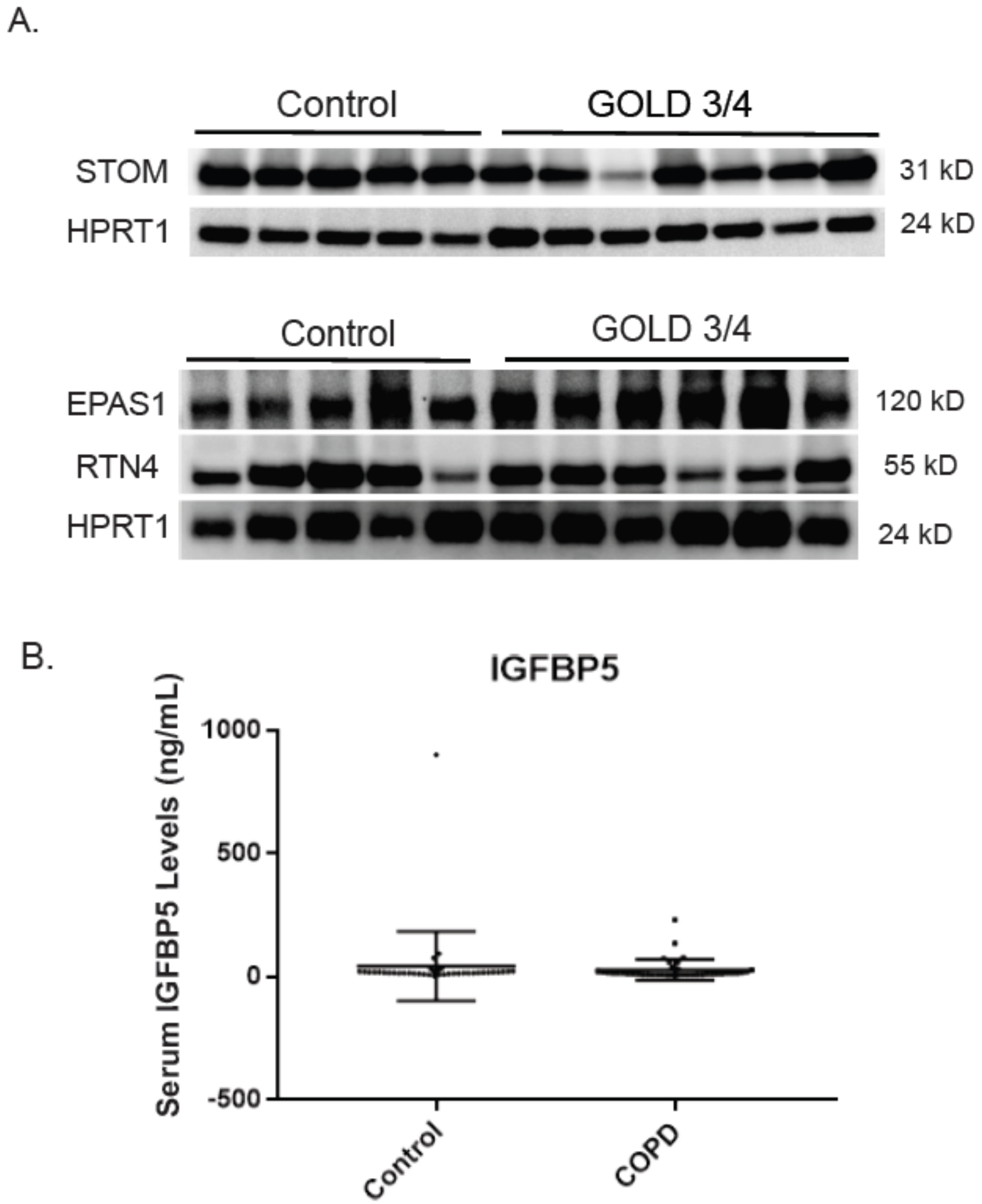
A.
Control
GOLD 3/4
STOM
31 kD
HPRT1
24 kD
Control
GOLD 3/4
EPAS1
120 kD
RTN4
55 kD
HPRT1
24 kD
B.
IGFBP5
Serum IGFBP5 Levels (ng/mL)
1000
500
0
-500
Control
COPD

Figure S7
